# Parent-report health-related quality of life in school-aged children with cerebral palsy: A cross-sectional study

**DOI:** 10.3389/fresc.2022.1080146

**Published:** 2022-12-05

**Authors:** Fábio Vila-Nova, Sofia Santos, Raul Oliveira, Rita Cordovil

**Affiliations:** ^1^ Faculdade de Motricidade Humana, Universidade de Lisboa, Lisboa, Portugal; ^2^ UIDEF, Instituto de Educação, Universidade de Lisboa, Lisboa, Portugal; ^3^ Neuromuscular Research Lab, Faculdade de Motricidade Humana, Universidade de Lisboa, Lisboa, Portugal; ^4^ CIPER, Faculdade de Motricidade Humana, Universidade de Lisboa, Lisboa, Portugal

**Keywords:** parent-report, health-related quality of life, children, adolescent, cerebral palsy

## Abstract

Quality of life is both a goal and an outcome in Cerebral Palsy (CP) rehabilitation. Children with CP may show impaired health-related quality of life (HRQoL) compared to their typical peers. Parents' perceptions of HRQoL of their children could help rehabilitation professionals to identify areas for intervention aiming to improve health and wellbeing. This study aims to compare the proxy HRQoL of Portuguese school-aged children with CP and the general population, and to analyze child and family correlation. Differences were examined using European normative data for children from 8 to 18 years. Correlation and regression analysis examined the association between child and family variables in the CP group with statistically significant low scores. Sixty-eight parents of children and adolescents with CP (12.5 ± 2.91 years) answered the KIDSCREEN-52 parent version. We identified clinically significantly lower HRQoL in four out of ten HRQoL domains (*Physical well-being, Autonomy, Moods & Emotions*, and *Bullying*) than the norm peers. Correlations were found between the number of siblings and *Autonomy* (r = .315), meaning that having more siblings was associated with greater autonomy, and between mobility and *Moods & Emotions* (r = −.261), where children with impaired mobility shown low scores as perceived by their parents. Age, sex, mobility and cognitive impairment explained 32% of *Physical well-being* scores (*p* < .001). Mobility and cognitive impairment explained 16% of *Bullying* scores (*p* = .001). Although the family and child variables identified in this study are non-modifiable, they can help in the identification and early intervention aimed at improving HRQoL. Rehabilitation professionals should assess parent perceptions, extending the HRQoL assessment to children who can report and other informants, aiming at fostering wellbeing in children and adolescents with CP.

## Introduction

Cerebral palsy (CP) is the most common physical disability in childhood. CP is a non-progressive neurodevelopmental disorder caused by impairments in the developing brain structure and function (1). The impaired motor function may be associated with intellectual disability, epilepsy, communication, and neuromuscular development, among others (1). The overall rates of associated impairments and functional limitations show that three in four children with CP are in pain; one in two have an intellectual disability; one in three cannot walk; one in four cannot talk; one in four have epilepsy; one in four have a behavior disorder; and one in five have a sleep disorder (2). The interaction with of associated impairments could lead to activity limitations and participation restrictions, influencing quality of life (QoL) in this population (3, 4).

In CP pediatric rehabilitation, QoL is a main goal in the longitudinal follow-up, also considered a primary outcome of intervention (5). The World Health Organization (WHO) defines QoL as a multidimensional construct, defined as individuals' holistic wellbeing perceptions of their position in life, embedded in a cultural, social, and environmental context (6). The Health-related quality of life (HRQoL) is considered a component of general QoL, and has been used to describe the biopsychosocial health of the patient, excluding other life domains, as perceived by the person or other individuals (7, 8).

Generic and specific instruments were developed and validated to assess HRQoL in CP pediatric population (9, 10). One limitation of HRQoL measurement is the difficulty in assessing children who cannot self-report due to intellectual, communication, or behavioral limitations. In these cases, the use of the parental report can provide information about children with CP, overcoming the difficulty of self-report ratings. Although the proxy reports may differ from the child's (11, 12), the parent perspective is a core element of the family-based approach in CP rehabilitation. Parents can provide relevant information on child's life when the information cannot be obtained directly from the child, being an active partner in family-based interventions (13).

Several studies examined the HRQoL as perceived by the children and youth with CP and their parents (14–16), and some assessed the congruence between parent and child reports within a multi-informant strategy (11, 12, 23). Most studies with parents and children, who can report, indicate a variation in agreement, with parents tending to underestimate scores (11, 23). Longo et al. (12) argues that children with CP tend to emphasize what they can do rating themselves at the highest level. In contrast, parent's perceptions could reflect a disability-related perspective emphasizing what the child cannot do. The differences with peers without disabilities or with other health conditions (17–19), and child, family and environmental predictors (20–22) have also been analyzed. Patients and parents in the CP clusters reported the lowest overall HRQOL in a comparison study with healthy children and those with obesity, asthma, cancer, diabetes, psychiatric disorders, and rheumatologic, gastrointestinal, cardiac and renal conditions (17). In addition, a systematic review of studies with children and youth with CP from low- and middle-income countries showed significantly poorer HRQoL on all instrument dimensions compared to age-matched controls and almost dimensions when compared to peers from high-income countries (24).

In proxy studies, low HRQoL in *Physical well-being*, *Social Support & Peers*, *School functioning* and *Autonomy* domains were previously identified (12, 14, 19, 21). Results may vary depending on the children's profile included in the studies. The SPARCLE HRQoL proxy study (21) with a European cohort of 551 adolescents with CP (316 boys, 235 girls) examined changes and predictors between childhood and adolescence. The authors highlight that childhood HRQoL was a consistent predictor of adolescent HRQoL. The results indicate a decrease in scores in six domains in the transition to adolescence (−1.3 to −3.8 points; *p* < 0.01). Furthermore, the authors identified a stable trend in the low scores reported by parents in childhood in *Physical well-being* when moving into adolescence (21). These findings reinforce the need for monitoring the HRQoL proxy report during the transition from childhood to adolescence, and later into adulthood. Age, motor function, IQ, communication, pain, psychological problems and parenting stress were identified as predictors of proxy HRQoL (12, 14, 19, 21).

Previous research on HRQoL in Portuguese children with CP examined psychological correlates and outcomes (25, 26), the parent caregiving burden (27), and prosocial behaviors outcomes within children with chronic health conditions, including CP, obesity, epilepsy and asthma and healthy controls (27, 28). However, majority of these studies included children who can self-report (IQ > 70). In addition, Portuguese children did not participated in the large studies within European CP population (21). For those with communication and cognitive impairment, the parent report could help to support intervention planning in rehabilitation programs. Assessment of child and family variables related to HRQoL as perceived by Portuguese parents, as well as the comparison with normative population can also provide useful information for health professionals.

Thus, the aim of this study is to (i) describe the perceptions of parents of Portuguese school-aged children with CP aged 8 to 18 years regarding their child HRQoL; (ii) identify differences in the HRQoL between this sample and the normative population; and (iii) examine relationships between child and family variables in clinically significant low HRQoL domain(s) in the group of children with CP, in order to identify areas for interventional studies in this population. We hypothesize that the differences between the CP group and the norm are similar to the findings of previous proxy studies with European CP population.

## Material and methods

This descriptive and exploratory cross-sectional study assessed data from a participation study with Portuguese children with CP (29). The research was approved by the Ethics Board of the Faculty of Human Kinetics (University of Lisbon), the Ethics Commission of Centro de Medicina de Reabilitação do Alcoitão, and rehabilitation centers directors. All parents gave written informed consent prior assessment.

### Participants

Rehabilitation centers in Portugal were invited to collaborate in identifying and inviting volunteers. The inclusion criterion was children and adolescents (8–18 years) with a medical diagnosis of CP according to European CP surveillance program (23). Exclusion criteria were having a severe intellectual disability (IQ < 50), a botulinum toxin injection in the last six months, or orthopedic surgical intervention in the last twelve months. Initially, 98 participants met the inclusion criteria in the region of Lisboa and Vale do Tejo, Beja and Faro. However, 29 parents were unable to participate due to lack of time, other activities of the child, and incompatibility of parents' schedules.

### Instruments

#### KIDSCREEN-52 parent version

The KIDSCREEN-52 is a cross-cultural and standardized generic instrument designed to measure HRQoL in children and adolescents aged 8–18 years with and without chronic health conditions, including asthma, epilepsy, cerebral palsy and obesity, among others (4). The 52-item questionnaire record information directly from the children (self-report), or through their parents (proxy report) on ten domains of QoL: *Physical well-being, Psychological well-being, Moods and Emotions, Self-perception, Autonomy, Parent Relation, Financial Resources, Social Support and Peers, School Environment*, and *Bullying* (30).

The KIDSCREEN-52 questionnaire was developed based on a literature review, expert consultation, and focus groups across Europe (4). The proxy report Portuguese version has good psychometric properties, with overall internal consistency (Cronbach's α) mean value of 0.82, varying between 0.64 (*Self-perception*) and 0.87 (*Financial resources*), similar to the countries involved in the instrument development and determining normative values (31). Rasch measurement properties of KIDSCREEN-52 are valid in a similar way in children with CP and in the general population (32). Comparisons of quality of life between such children are therefore valid.

For the score calculation, raw data were summed scaled to yield a score in the range 0–100, with higher scores indicating a higher HRQoL. The results can also be converted in T-scores with the provided syntax. Comparison with the European normative population can be made by identifying where a T-score domain mean is in the threshold around the norm. The mean score below or above the threshold indicates a clinically significant low or high HRQoL, respectively, in the specific domain (30).

#### Clinical and socio-demographic profile

Parents of children with CP reported information about their child mobility in school (with or without assistive devices), and family variables, number of siblings, family type (single-parent/two-parent), and mother education level (basic literacy to higher education) in a study form. Information on participant's age, sex, CP type, motor and cognitive impairment was recorded according to the clinical information provided by rehabilitation centers. For cognitive impairment, participants were described as none or mild (IQ ≥ 70) and moderate (50 > IQ < 70). The gross motor function was described using the Gross Motor Function Classification System Expanded and Revised (GMFCS-ER), a valid and reliable 5-level categorization system for children and youth with CP, from level I (independent walking in all environments) to level V (transported in a manual wheelchair or powered mobility in all environments) (33).

### Procedures

Parents and children with CP were invited to participate through the rehabilitation centers that agreed to collaborate with the study. Volunteers were invited by telephone or directly at the rehabilitation service, the objectives and procedures of the study were explained. For those who agreed to participate, an appointment was scheduled according to the family's' availability. The rehabilitation professionals provided clinical information in a study form. The parents answered the KIDSCREEN-52 and provided socio-demographic information. In this study, the proxy version was used to record parent perceptions on HRQoL of children with CP due to the inclusion of participants with moderate cognitive impairment (70 < IQ > 50), the time constraint to assess the readiness of children with mild cognitive impairment to self-report, and the relevance of parent-reported information in family-based intervention.

### Statistical analysis

Descriptive statistics was used to characterize the sample and KIDSCREEN-52 summative scores and T-scores per domain. The calculation of the summative scores was performed and then transformed into T-scores. Parent proxy normative data from European children and adolescents aged 8 to 18 were extracted from the manual. We assessed the magnitude of differences in summative scores between groups with independent t-tests in all ten domains, with statistical significance when *p* < 0.01 due to multiple comparisons. The Pearson Product Moment was used to identify associations between child functioning (age, sex, mobility in school, cognitive impairment) and family variables (number of siblings, mother education level, family type) in domains with statistically significant low scores. Linear regression examined significant predictors when two or more variables showed significant correlation with a domain. To determine whether participants with CP have lower or higher HRQoL to the norm, we performed the author's proposed calculation, where a mean score below or above the threshold indicates a clinically significant difference in HRQoL in the domain. These differences were confirmed when the effect size was >0.5. Statistical analyses were performed using the SPSS 24.0 version software program.

## Results

The analysis included proxy report from 68 children and adolescents with CP (8–18 years; mean age 12.71, SD: 2.94), since one questionnaire had missing data and was excluded. Sixty-six percent of participants were boys, 41.2%% uses an assistive device for mobility in school (walker, wheelchair or electric wheelchair), and 66.2% had none to mild cognitive impairment. Almost 90% of the participants have at least one sibling, and 70.6% lives with two parents, with 42.7% from mothers with basic or elementary education (Table 1). The majority of respondents (95%) were mothers (*n* = 65), followed by fathers (*n* = 2) and grandfather (*n* = 1).

**Table 1:** Participants profile (*n* = 68).

Variable		*N* (%)
Age	Mean: 12.71 years; SD: 2.94	
	8–12 year	31 (45.6)
	13–18 year	37 (54.4)
Sex	Male	45 (66.2)
	Female	23 (33.8)
Cognitive impairment	None	14 (20.6)
	Mild	31 (45.6)
	Moderate	23 (33.8)
Gross Motor Function	Level I	33 (48.5)
	Level II	10 (14.7)
	Level III	12 (17.6)
	Level IV	7 (10.3)
	Level V	6 (8.8)
Mobility in school	Without assistive device	40 (58.8)
	With assistive device	28 (41.2)
Mother education level	Higher education	20 (29.4)
	Secondary education (12th grade)	19 (27.9)
	Elementary education (9th grade)	21 (30.9)
	Basic literacy	8 (11.8)
Family type	Two-parent	48 (70.6)
	Single-parent	20 (29.4)
Siblings	None	8 (11.8)
	1	31 (45.6)
	2	17 (25.0)
	3+	12 (17.6)

Table 2 show means and standard deviations for summative and T-scores from parents of children with CP and normative population. KIDSCREEN-52 proxy summative mean scores ranged from 57.42 (SD: 17.48) in the *Physical well-being* domain to 78.12 (SD: 15.43) in the *Parent relation* domain. Parametric tests show significant differences with norm in five out of ten domains. Summative scores were lower for CP group in the *Physical well-being, Moods & Emotions, Autonomy, Social Support and Peers*, and *Bullying* domains (*p* < .001). The T-Scores comparison shown clinically significant low HRQoL in the *Physical well-being* (*d *= 0.85)*, Moods & Emotions* (*d *= 1.25)*, Autonomy* (*d *= 0.61), and *Bullying* (*d *= 1.00) (Table 2).

**Table 2:** Summative and T-Scores comparison between proxy HRQoL in Portuguese children with CP (*n* = 68) and European norm (*n* = 15,485–15,897).

HRQoL domains	Sum Scores			T-Scores		
	*CP M (SD)*	*Norm M (SD)*	*p*	*CP M (SD)*	*Norm M (SD)*	*d*
*Physical well-being*	57.43 (17.48)	72.08 (17.08)	<0.001	42.12 (8.85)^a^	49.98 (10.0)	0.83
*Psychological well-being*	75.80 (13.85)	74.94 (15.4)	0.646	50.40 (9.57)	49.99 (10.0)	0.04
*Mood & emotions*	66.86 (10.79)	81.38 (13.4)	<0.001	39.77 (5.69)^a^	49.99 (9.99)	1.25
*Self-perception*	71.99 (15.23)	76.72 (16.8)	0.020	46.85 (8.36)	49.99 (10.0)	0.34
*Autonomy*	63.97 (18.53)	75.32 (18.0)	<0.001	44.05 (9.33)^a^	50.01 (10.0)	0.61
*Parent relation*	78.13 (15.42)	77.70 (15.6)	0.821	50.14 (9.71)	50.00 (10.0)	0.01
*Social Support & Peers*	59.93 (20.67)	67.93 (18.0)	<0.001	45.76 (11.44)	49.99 (10.0)	0.39
*School environment*	73.78 (12.36)	69.43 (17.9)	0.045	51.87 (7.18)	49.99 (10.0)	0.21
*Bullying*	70.59 (20.08)	88.43 (15.2)	<0.001	38.96 (11.87)^a^	50.00 (9.99)	1.00
*Financial resources*	68.63 (23.71)	66.71 (25.6)	0.212	50.69 (9.50)	50.00 (10.0)	0.07

^a^ Clinically significant. CP, cerebral palsy; SD, standard deviation.

Correlation analysis between child and family variables in the statistically significant low HRQoL domains indicate small to medium correlation between the *Physical well-being* scores with age (r = −.321; *p* < .001), sex (r = −.234; *p* = .002), cognitive impairment (r = −.378; *p* = .001), and mobility (r = −.496; *p* < .001). The *Autonomy* score was correlated with the number of siblings (r = .315; *p* = .009). Mobility (r = −.261; *p* = .03) was correlated with *Moods & Emotions* domain. In the *Bullying* domain, small to medium correlation was also found with mobility (r = −.260; *p* < 0.05) and cognitive impairment (r = −.418; *p* < .01). We did not found significant correlations between child and family variables with *Social Support and Peers* domain.

The regression analysis shown age (*p* = .512), sex (*p* = .01), mobility (*p* = .001) and cognitive impairment (*p* = 0.09) explaining 32% of HRQoL variance for the *Physical well-being* domain adjusted *R*^2 ^= .319, F(4, 63) = 8.85, *p* < .001 (Table 3). The regression analysis for *Bullying* revealed mobility (*p* = .394) and cognitive impairment (*p* = .003) explaining 16% of scores, *R*^2 ^= .159, F(2, 65) = 7.33, *p* = .001.

**Table 3:** Summary of the regression model for *Physical wellbeing* scores.

	*B*	*SE B*	*ß*	*t*	*R²*	*Adjusted R²*
					.360	.319
Age	*−* *.* *413*	*.* *626*	−0.69	−.659		
Sex	*−9* *.* *873*	*3* *.* *702*	−.269	−2.667*		
Mobility	14.321	3.989	.406	−1.720**		
Cognitive impairment	−7.028	4.087	−.192	3.590		

* *p *< .05.

** *p* ≤ .01.

Sex (Male = 1; Female = 0); Cognitive impairment (IQ ≥ 70 = 1; 50 > IQ < 70 = 0); Mobility in school (without assistive device = 1; with assistive device = 0).

## Discussion

Parents' perception provides relevant information in family-based pediatric rehabilitation and may contribute to identify areas for intervention throughout the follow-up of the child with CP. In this study, we examined the differences in proxy HRQoL of Portuguese school-aged children with CP and the norm for the European population. We found differences with clinically significant low HRQoL in four out of ten domains of the KIDSCREEN-52 in children with CP aged 8 18 years (*Physical well-being, Autonomy, Moods & Emotions,* and *Bullying*). The correlation analysis shown that parents who have other children reported high *Autonomy* scores for their children with CP, and those with children with impaired mobility reported low scores in *Moods & Emotions*. Age, sex, mobility and cognitive impairment explained 32% of *Physical well-being* scores. Mobility and cognitive impairment explained 16% of *Bullying* scores. Our findings are similar to the proxy studies conducted in the CP population in Europe (11, 14, 18, 19).

### Physical well-being

The findings of low *Physical well-being* and association with poor motor functioning are supported by extensive previous research (11, 16, 20, 34). HRQoL low scores regarding performance of habitual physical activity (PA), mobility and physically demanding sport and play activities are frequently reported by both parents and children with CP (16). Previous studies shown that children with greater mobility impairment have less opportunities of leisure PA participation, being at risk of not experiencing health benefits of PA, ultimately influencing physical wellbeing (24, 35). Parental awareness about motor impairment of children may influence the lower scores. The stability in parental perceptions regarding physical wellbeing in the transition to adolescence makes it important to promote this aspect from childhood (21). Verschuren et al. (36) underlines the importance of monitoring PA and movement behavior of children and youth with CP, in clinical routine, considering also pain, sleep, and sedentary time. Based on the International Classification of Functioning, Disability and Health, Rosenbaum et al. (37) proposes, as ingredients of pediatric rehabilitation, the life-course promotion of physical wellbeing aimed at improving function in activities that are fun, and of interest to the child, with his family and friends, in all possible environments.

### Autonomy

In KIDSCREEN, *Autonomy* domain refers to the child's ability to give his/hers opinion and make choices in free time, and whether they are sufficiently provided with opportunities to participate in social and recreational activities (9). Although Rapp et al. (21) identified the number of siblings as predictors for psychological wellbeing scores in adolescents with CP; we found correlation with the *Autonomy* scores. In our study, parents with other children reported higher scores in the *Autonomy* domain of their child with CP. In a qualitative study with female adults with CP that examined families and individual family member's contribution to overall QoL, one emergent theme was the importance of siblings as friends, mentors, instructors, and supporters in interaction during their time together in everyday life situations, including play (38). This interaction between siblings seems to reflect positively on parents' perceptions of their child autonomy, due to the opportunities to interaction and choices making with others. A large family can also minimize the burden on parents regarding the care of their child with disabilities in everyday life activities (39). Healthy relationships with siblings in childhood can extend into adult life, positively influencing the QoL of a person with CP (38). Engagement of siblings in rehabilitation follow-up may allow us to hear their perceptions about family everyday life situations; questions about the diagnosis, intervention, follow-up; and as other informant about the QoL of the sibling with CP (13, 39).

### Moods & emotions

We identified low HRQoL in the *Moods & Emotions* domain from the perspective of parents of children with CP when compared to norm. In an agreement study with KIDSCREEN-52 among Spanish parents and children with CP, Longo et al. (12) found that parents reported lower values than their child in this domain, with poor correlation between them. The discrepancies are greater in domains related to psychological wellbeing, and that address more abstract situations such as depressive moods and emotions and stressful feelings (16, 40). In a study with Portuguese children with CP with mild cognitive impairments, Frontini et al. (25) identified that higher HRQoL was significantly related to lower levels of psychopathological symptoms, higher levels of prosocial behavior and a lower need for social activities. In this study, children with adjustment problems seem to be especially at risk of decreased social support provided by social activities, highlighting the relevance of promoting these activities, in particular for children with CP with more adjustment difficulties. These findings reinforces the importance of monitoring the emotional and psychological aspects of children with CP (21, 25). This may partly explain why we did not have found correlations with the variables analyzed in our study.

### Bullying

In contrast with Rapp et al. (21), we found lower HRQoL in the *Bullying* domain as perceived by parents of children with greater cognitive impairment. This dimension explores both the feeling of being rejected by others as well as the feeling of anxiety toward peers related to bullying (9). This situation is represented by the perpetration of annoying, inappropriate, and unpleasant behaviors by peers in the school environment, which may be repeated and present an imbalance of verbal, physical, or psychological power (9, 41). In a study with a representative sample of European children and adolescents aged 8 to 18 years, researchers found that 20.6% had experienced bullying, with a higher risk in those with chronic health conditions (41). Stang et al. (42) identified in a qualitative study with 43 children with CP without cognitive impairment that bullying was more frequent and physical in children in the GMFCS I, II & III. In children in GMFCS IV and V, the frequency was lowest, inferring that in this case, the severity of disability may play a role in the amount of bullying experienced (42). Proxy reporting on experiences in the school environment may be biased by the parents’ own perception of their children functioning. Although children with CP report bullying to their parents, we did not assess whether this information was reported by their children or by school staff. Thus, health and education professionals should be aware to the child's experiences on peer behaviors and social acceptance in school environment. The communication between family, school and rehabilitation team could promote early identification and intervention to promote resilience and minimize the effects of bullying during school years (13, 42).

### Social support and peers

Finally, we found statistical differences in the *Social Support and Peers* domain between the sample of Portuguese children with CP and the norm, but without clinical significance. Similarly, Longo et al. (12) identified low scores of Spanish parents and children with CP in this domain, with strong correlation between parent and child report. In contrast, Boldyreva et al. (2020) found high scores in adolescents with CP in this domain in a comparison study with participants with CP, CP and epilepsy, epilepsy alone and norm population, with clinically significant lower HRQoL only for the epilepsy group. Vles et al. (14) propose that low HRQoL on the *Social Support & Peers* domain may be related to concerns of the caregivers rather than the child experience. Although the differences were not clinically significant, one should monitor peer relations and social participation. Participation studies indicate that involvement in social activities is high (29). However, with greater involvement with peers and other people beyond family members in children with high physical, cognitive and communication functioning, as well as in those with parents with high education levels (43).

### Limitations and future directions

This study has limitations that must be considered. We assessed a small convenience sample of children with CP, which excluded those with severe intellectual impairment and those who underwent recent orthopedic interventions. The HRQoL is a multidimensional construct without a consensual definition and, in this study, was assessed mostly from the mother's perspective. We did not collect other data from the respondent such as family income and parents' routines regarding work and childcare that could influence the results. Moreover, multiple informants (father, caregiver, siblings, other family-members, and teachers) could broad the perspective of HRQoL of children who cannot report. We found associations between child and family variables in low HRQoL domains. However, studies with a larger sample may confirm these findings in the Portuguese population with CP. Although we have not analysed psychological variables, previous studies reinforce the need to implement the follow-up of psychological wellbeing in the clinical routine with children with CP and their families (25). Future research could examine bullying reports in Portuguese children with physical disabilities (44). Finally, the role of siblings in the family ecology could be examined in Portuguese children with CP.

## Conclusion

Parents of children with CP perspectives may indicate areas for pediatric rehabilitation intervention. We identified clinically significant lower HRQoL in *Physical well-being, Autonomy, Moods & Emotions*, and *Bullying* domains in Portuguese children with CP aged 8 to 18 years when compared to the norm population. The child and family variables identified in this study may help health professionals to HRQoL monitor and intervene throughout the development of school-aged children with CP. Importantly, when possible, assess the child report and others informants to identify similarities or discrepancies with the parent report, thus adjusting the intervention aimed at fostering HRQoL in this population.

## Data Availability

The raw data supporting the conclusions of this article will be made available by the authors, without undue reservation.
